# Novel approach to soft tissue regeneration: *in vitro* study of compound hyaluronic acid and horizontal platelet-rich fibrin combination

**DOI:** 10.1590/1678-7757-2023-0294

**Published:** 2024-05-10

**Authors:** Yun QIU, Kailun SHEN, Hongjiang WEI, Yufeng ZHANG, Yulan WANG, Yi BAI

**Affiliations:** 1 Wuhan University School & Hospital of Stomatology Hubei Key Laboratory of Stomatology Wuhan China Wuhan University, School & Hospital of Stomatology, Hubei Key Laboratory of Stomatology, State Key Laboratory of Oral & Maxillofacial Reconstruction and Regeneration, Key Laboratory of Oral Biomedicine Ministry of Education, Wuhan, China.; 2 Stomatological Hospital of Xiamen Medical School Xiamen China Stomatological Hospital of Xiamen Medical School, Xiamen, China. University of Wuhan, School and Hospital of Stomatology, Department of Dental Implantology, Wuhan, China.; 3 University of Wuhan School and Hospital of Stomatology Department of Dental Implantology Wuhan China; 3 Wuhan University School of Medicine Medical Research Institute Wuhan China Wuhan University, School of Medicine, Medical Research Institute, Wuhan, China.

**Keywords:** Soft tissue regeneration, Hyaluronic acid, Horizontal platelet-rich fibrin, Gingival fibroblast

## Abstract

**Objective:**

This study aims to develop a compound biomaterial to achieve effective soft tissue regeneration.

**Methodology:**

Compound hyaluronic acid (CHA) and liquid horizontal-platelet-rich fibrin (H-PRF) were mixed at a ratio of 1:1 to form a CHA-PRF gel. Human gingival fibroblasts (HGFs) were used in this study. The effect of CHA, H-PRF, and the CHA-PRF gel on cell viability was evaluated by CCK-8 assays. Then, the effect of CHA, H-PRF, and the CHA-PRF gel on collagen formation and deposition was evaluated by qRT‒PCR and immunofluorescence analysis. Finally, qRT‒PCR, immunofluorescence analysis, Transwell assays, and scratch wound-healing assays were performed to determine how CHA, H-PRF, and the CHA-PRF gel affect the migration of HGFs.

**Results:**

The combination of CHA and H-PRF shortened the coagulation time of liquid H-PRF. Compared to the pure CHA and H-PRF group, the CHA-PRF group exhibited the highest cell proliferation at all time points, as shown by the CCK-8 assay. Col1a and FAK were expressed at the highest levels in the CHA-PRF group, as shown by qRT‒PCR. CHA and PRF could stimulate collagen formation and HGF migration, as observed by fluorescence microscopy analysis of COL1 and F-actin and Transwell and scratch healing assays.

**Conclusion:**

The CHA-PRF group exhibited greater potential to promote soft tissue regeneration by inducing cell proliferation, collagen synthesis, and migration in HGFs than the pure CHA or H-PRF group. CHA-PRF can serve as a great candidate for use alone or in combination with autografts in periodontal or peri-implant soft tissue regeneration.

## Introduction

Soft tissue grafting is becoming widely used in dental practice.^1^ At first, it was used only to increase gingival thickness or keratinized tissue width around natural teeth or peri-implants to maintain the periodontal tissue health of natural teeth or implants.^2^ With further development of dental medicine, this process is now also widely used for the treatment of gingival recession of natural teeth or peri-implants.^3,4^ With the growing interest in oral aesthetics, soft tissue grafting is also being used for gingival papilla reconstruction, gingival thickening prior to orthodontic tooth movement, and implant abutment masking.^1,5^

Currently, the most used material for soft tissue augmentation in clinical practice is autologous soft tissue grafts, which mainly include free gingival grafts (FGGs) and connective tissue grafts (CTGs) collected from the patient’s palate or maxillary tuberosity area. Although autologous grafts have shown great outcomes, there are various challenges, such as limited donor areas, donor site pain, high technical sensitivity, long surgery time, and postoperative bleeding. Therefore, there is high demand in clinical practice for soft tissue substitutes made of biomaterials.

Recently, platelet-rich fibrin (PRF) has been widely used as an autologous biomaterial in periodontal tissue regeneration. PRF contains the three essential elements for tissue regeneration: a fibrin three-dimensional scaffold, viable cells, and various growth factors. Growth factors have been shown to slowly release from PRF over 10-day periods, which endowed PRF with sustained regeneration capacity.^6^ PRF can effectively stimulate the proliferation and differentiation of gingival cells, thereby promoting soft tissue healing. Liquid PRF can be injected into the periodontium to improve the gingival biotype and promote gingival papilla regeneration.^7^Although PRF has shown promising results on soft tissue wound healing in a systematic review,^8^ its regenerative capacity is still inferior to that of autografts, and further efforts are needed to enhance its activity. Horizontal-PRF(H-PRF) was a novel kind of PRF that was prepared by horizontal centrifugation. Studies suggest that H-PRF yields a more uniformly distributed product with higher concentrations of viable cells and better separation of red blood cells compared to PRF prepared by fixed-angle centrifugation, which makes it a potential candidate for soft tissue regeneration.^9^

Hyaluronic acid (HA) has received much attention in promoting periodontal regeneration in recent years. HA is an anionic, nonsulfated glycosaminoglycan that is widely present in all living organisms, especially in the extracellular matrix and soft connective tissue.^10^ Compound hyaluronic acid (CHA) is a combination of hyaluronic acid and a small amount of active compounds including vitamins, mineral salts, antioxidants, and other compounds. It has been widely used to stimulate the proliferation of fibroblasts and increase the formation of collagen. CHA not only presents a suitable microenvironment for fibroblasts but also stimulates their biosynthetic capacity.^11^ Some clinical studies have proven the efficacy of CHA in the treatment of papilla deficiencies.^12,13^ The hypothesis of this study is that the combination of CHA and H-PRF can further promote periodontal tissue, and the aim was to evaluate its effect on HGFs *in vitro*.

## Methodology

The study protocol was approved by the institutional review board of School and Hospital of Stomatology, Wuhan University (B52/2020).

### Preparation of liquid H-PRF and the culture medium

This study was conducted with volunteers’ informed consent obtained before blood collection. Blood samples from healthy volunteers were collected in PET plastic tubes (Plasmatrident, Weiyin Technology Co., Ltd., Wuhan, China) and subjected to horizontal centrifugation at 500 × g for 8 min (Plasmatrident, Weiyin Technology Co., Ltd., Wuhan, China). The upper yellow layer containing liquid H-PRF was collected by a syringe. H-PRF and CHA (HA135, Filorga) were used alone or in combination at a ratio of 1:1. After solidification (except for pure CHA), 5 mL of Dulbecco’s Modified Eagle Medium (DMEM) was added to 1 mL of PRF, CHA, and CHA-PRF, soaked for three days. Then, the DMEM was collected as conditioned medium. The conditioned medium was added to the culture medium at a proportion of 20% in later experiments, following previous studies.^14,15^

### Coagulation time test

Liquid H-PRF alone or in combination with CHA at a ratio of 1:1 was loaded into the syringe. At each timepoint, a drop of liquid was pushed out of the syringe. When the syringe was blocked, the time was noted as coagulation time. The experiment was repeated three times.

### Cell culture

Fresh gingiva was obtained from the extracted wisdom teeth of volunteers with ethical approval. The gingiva was washed with PBS supplemented with 10% penicillin‒streptomycin solution (Gibco) and cut into 1 mm^3^pieces. The tissue was allowed to attach to the bottom of a T25 culture flask for 4 h in DMEM supplemented with 10% fetal bovine serum (FBS). Then, the T25 culture flask was turned upside down and cultured in a 37 °C humidified incubator with 5% CO_2_. When the cells were nearly confluent, they were passaged using trypsin digestion. The cells used in this study were between passages 2 and 7.

### Cell proliferation assay

A Cell Counting Kit (CCK-8, Biosharp) assay was used to assess the proliferation of HGFs. A total of 10^3^ cells per well were inoculated into a 96-well plate, and 100 µL of culture medium containing 20% CHA, PRF, or CHA-PRF soaking liquid was added to each well. Then, 10 µL of CCK-8 solution was added to each well after 1, 3, 5 and 7 days of incubation. The cells were incubated with the CCK-8 solution for 40 min, the supernatant was removed, and the absorbance was measured at 490 nm.

### Immunofluorescence analysis

HGFs were cultured with 20% CHA, PRF, or CHA-PRF soaking liquid for seven days and then fixed with 4% paraformaldehyde for 15 min. The cells were washed three times with PBS and permeabilized with 0.5% Triton X-100 at room temperature for 20 min. After that, the cells were washed three times with PBS and blocked with 1% BSA solution for 1 h. Then, staining was performed by incubation at 4 °C overnight with primary antibodies (COL1A1, ABclonal, 1:200) and 1 h of incubation with 594 secondary antibodies (Alexa Fluor^®^ 594 goat anti-mouse IgG2a) at 37 °C. To visualize F-actin, 100 nM FITC-labeled phalloidin was added and incubated at 37 °C for 30 min. Finally, the cells were counterstained with DAPI for 5 min. Fluorescence images were obtained by an inverted fluorescence microscope (ZEISS).

### mRNA extraction and qPCR

**Figure 1:**
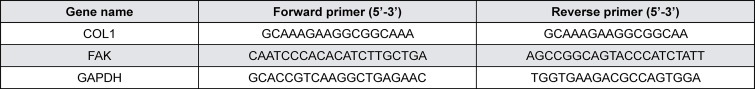
Primers’ sequences

HGFs were cultured as described above for seven days and washed three times with PBS. Total RNA was extracted by TRIzol reagent (Invitrogen) following manufacturer’s instructions. mRNA was reversely transcribed into cDNA using HiScript II Reverse Transcriptase (Vazyme). Then, real-time RT‒PCR was performed on a Roche LightCycler® 480 using Taq Pro Universal SYBR qPCR Master Mix (Vazyme). The PCR primer sequences are listed in Figure 1. The results were calculated by 2^-ΔΔCt.

### Scratch wound-healing assay

HGFs were seeded into 24-well plates and cultured until they reached 100% confluence. Labels were made on the bottom of the plate to mark the captured view, and scratches were made using the same pipette tip and a ruler. FBS-free culture medium was added to the plate according to the groups. Photos of the same view were taken under a microscope at 0, 24, and 48 h after the scratch was made. For each group, six views were included in the analysis. ImageJ was used to calculate the remaining area at each timepoint.

### Transwell migration assay

Migration measurements were performed using a Transwell plate with a pore size of 8 μm. HGFs were digested and resuspended in FBS-free DMEM at a concentration of 1×10^4^/mL. In total, 200 µL of the cell suspension was inoculated into the upper chamber of the Transwell. Culture medium containing FBS and 20% CHA, PRF, or CHA-PRF soaking liquid were added into the lower chamber. After 24 h and 48 h of incubation, the Transwells were fixed with 4% paraformaldehyde for 15 min. The cells in the upper chamber were carefully removed with cotton swabs, and the chamber was stained with crystal violet. After the chamber was washed three times with PBS, five fields of view were randomly captured under a microscope, and the number of cells was counted using ImageJ software.

### Statistical analysis

GraphPad Prism software version 8.0.2 was used to analyze the data. A normality test was performed before the differential analysis. A t test was performed to analyze two groups, and one-way ANOVA was conducted when the group number was higher (*P < 0.033, **P < 0.01, and ***P < 0.001).

## Results

### Coagulation time

**Figure 2:**
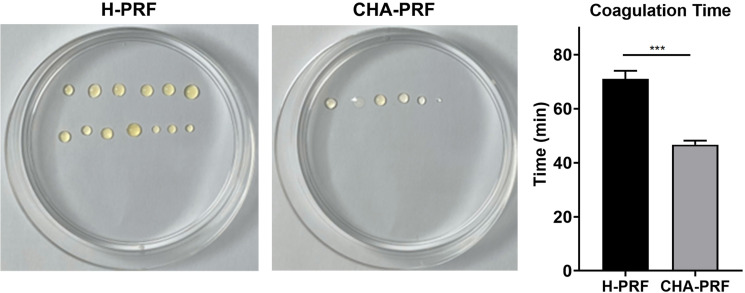
Coagulation time of H-PRF and CHA-PRF. ***P < 0.001

H-PRF was obtained by centrifugation, and the coagulation time was determined. This time provides a reference for clinical use that directly affects the clinical operation time. The average coagulation time was 71 min for pure liquid H-PRF and 46.7 min for the mixture of CHA and PRF (CHA-PRF). The color of H-PRF was yellow, and CHA-PRF was brighter, tougher, and more tensile (Figure 2).

### Cell proliferation

**Figure 3:**
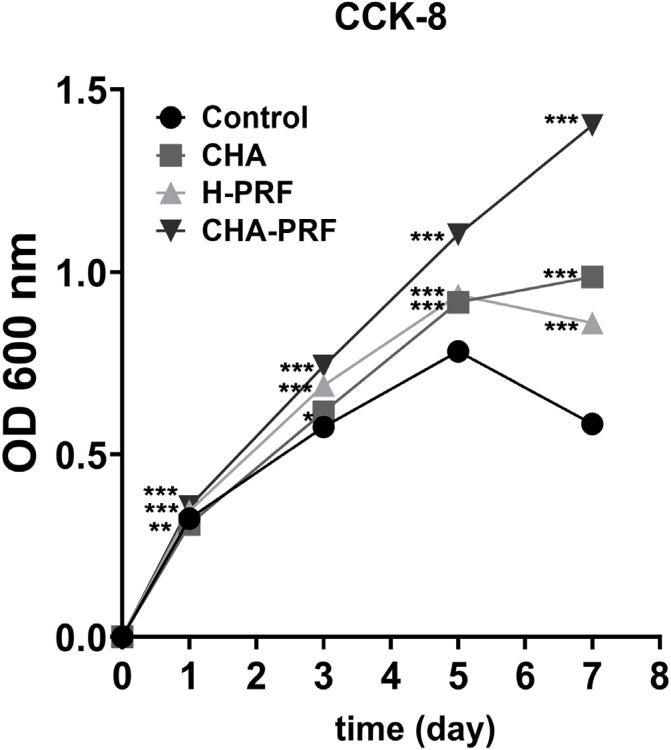
Cell proliferation ability of control, CHA, H-PRF, and CHA-PRF groups, tested by CCK-8. *P < 0.033, **P < 0.01, and ***P < 0.001

As shown in Figure 3 and Table 1, CHA and H-PRF exhibited excellent biocompatibility. CHA and H-PRF facilitated the proliferation of HGFs over seven days, and the cell density on Day 7 was higher than that in the control group, as observed by microscopy. The CHA-PRF group exhibited the highest cell viability from Day 1 to Day 7.

**Table 1:** Daily means of optical densities of groups tested by CCK8

	CHA	H-PRF	CHA-PRF
Day 1	0.95	1.06	1.10
Day 3	1.07	1.19	1.29
Day 5	1.17	1.19	1.41
Day 7	1.69	1.47	2.40

### Cellular collagen formation

**Figure 4:**
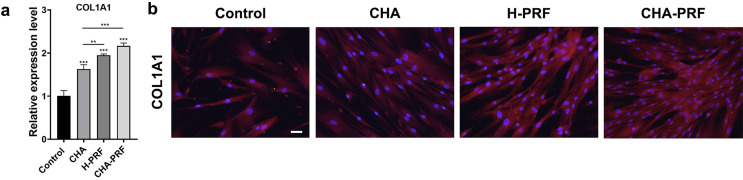
Collagen formation ability. a) Gene expression level of COL1A1 in control, CHA, H-PRF, and CHA-PRF groups. **P < 0.01, and ***P < 0.001. B) Fluorescence images of COL1A1 (red) and DAPI (blue) (scare bar, 100 um)

Type I collagen is the major component of the extracellular matrix (ECM) in periodontal tissues, and it acts as a signaling molecule to regulate cell proliferation, differentiation, and migration.^16^ As shown in Figure 4, the addition of CHA and H-PRF stimulated the gene expression of COL1A1, and COL1A1 gene expression was higher in the PRF group than in the CHA group. Moreover, the CHA-PRF group showed the highest expression level of COL1A1. The fluorescence images showed similar trends, and COL1A1 in the CHA-PRF group exhibited the highest fluorescence intensity. Thus, the addition of CHA and H-PRF could stimulate the formation of type I collagen, and CHA-PRF showed the best effect.

**Figure 5:**
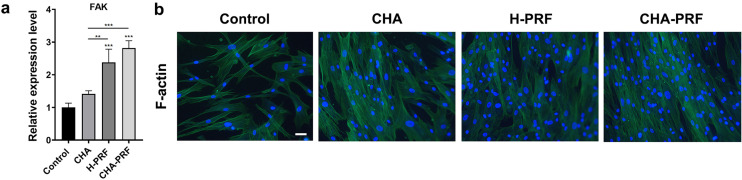
Cellular migrate ability. a) Gene expression level of FAK in control, CHA, H-PRF, and CHA-PRF groups. **P < 0.01, and ***P < 0.001. B) Fluorescence images of F-actin (green) and DAPI (blue) (scare bar, 100 um)

### Cytoskeleton changes

FAK plays an important role in cytoskeletal modifications, thereby accelerating cell migration.^17^ qRT‒PCR analysis revealed that H-PRF increased FAK mRNA expression in HGFs. However, the CHA group showed no differences in expression compared to the control group. The CHA-PRF group showed the highest FAK expression.

FITC-labeled phalloidin was used to stain cellular F-actin, which is a major component of the cytoskeleton and plays a critical role in cell migration.^18^ As shown in the fluorescence images, both CHA and H-PRF stimulated the reorganization of F-actin. The fluorescence signal in the cytoplasm was enhanced and arranged as bundles after the addition of CHA and H-PRF. This phenomenon was most obvious in the CHA-PRF group. Therefore, CHA and H-PRF can promote HGF cytoskeletal remodeling, which is related to the function and mobility of cells.

### Cellular migration

**Figure 6:**
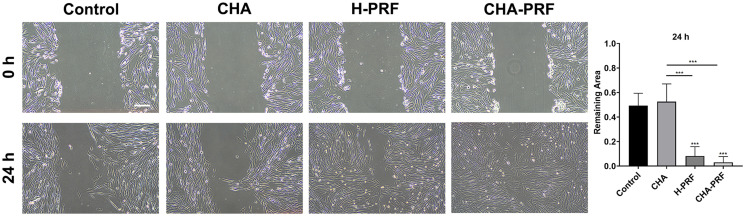
Scratches wound healing assay. A) images of 0 h and 24 h of HGF, same site (scare bar, 200 um). B) Calculated relative reaming area of control, CHA, H-PRF, and CHA-PRF group at 24 h compared to 0 h. ***P < 0.001

Scratches were performed in 24-well plates, and the HGFs were treated with culture medium containing 20% CHA, H-PRF, or CHA-PRF soaking liquid. When calculating the remaining area, it was found that the addition of H-PRF decreased the remaining area at a faster rate, but CHA showed the same effect as that in the control group. Among the four groups, the CHA-PRF group exhibited the best promotion of cell migration, and the HGFs almost filled the scratch at 24 h (Figure 6).

**Figure 7:**
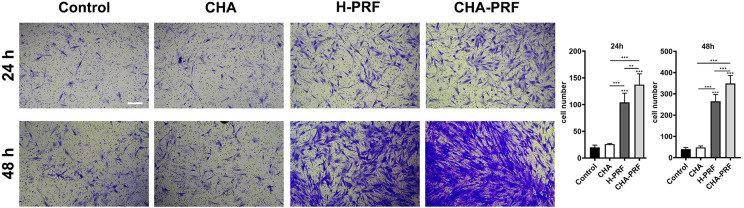
Transwell assay. A) images of 24 h and 48 h of the lower side of transwell chamber after crystal violet staining (scare bar, 200 um). B) cell counts of the lower side of transwell chamber, five views. **P < 0.01, and ***P < 0.001

Transwell assays were performed, and the soaking liquid of CHA, H-PRF, or CHA-PRF was used as a chemotactic agent and added to the lower chamber. The number of cells on the lower side of the Transwell chamber was increased in the PRF and CHA-PRF groups compared to the control group at 24 h and 48 h (Figure 7). However, CHA alone did not induce chemotaxis in HGFs.

## Discussion

The gingiva is composed of several components, including gingival cells (such as fibroblasts, epithelial cells, and immune cells), extracellular matrix (ECM), and neurovascular structures.^19^ Gingival fibroblasts play an essential role in maintaining the structural integrity and function of gingiva. HGFs can receive signals from growth factors and cytokines, thereby modifying cell structure and activity to respond to the surrounding environment.

Proliferation of HGFs was stimulated seven days after CHA or PRF treatment. The combination of CHA and PRF presented an even better effect. This might be due to the increased activation of platelets in H-PRF in response to CHA components, including mineral salt components such as calcium chloride.^20^ Serving as coagulation factor IV, calcium ions activate the endogenous clotting pathway and accelerate coagulation. Upon platelets activation, the contents inside α-granules were released and elevated the growth factors concentration. This can also explain the decreased coagulation time of H-PRF and CHA.

Our results corroborate a previous study in which researchers found that L-PRF and HA can both stimulate the growth of HGFs.^21^ Another study revealed that the combination of HA and PRF can increase human dermal fibroblasts activity and show better effects in combination.^22^

Additionally, the impact of compounds on cell migration and collagen production were thoroughly examined. HGFs possess the ability of direct site migration. These cells migrate to specific sites and mediate local tissue repair and regeneration. Migration requires cell deformation and changes in cell adhesion.^23^ To large degrees, the morphology change of HGFs contributes to the filamentous actin (F-actin) cytoskeleton. In HGFs, F-actin is linked to the ECM via transmembrane integrins and contains a variety of proteins. Integrins recognize extracellular matrix (ECM) and active molecules, activate focal adhesion kinase (FAK), and further regulate downstream cytoskeletal remodeling.^24^ FAK can be stimulated by TGF-β,^25^ which is one of the components of PRF. Additionally, PDGF and VEGF can increase HGF migration.^26^ CHA lacks chemokines that induce HGF migration, so in wound healing assay and transwell assays CHA exhibit the same effect as the control group. However, the addition of CHA increased the activation of platelets and cytokines release. This could explain why CHA-PRF has better chemotactic activity than pure PRF.

HGFs are mesenchymal in origin and play an important role in gingival ECM formation. These cells are responsible for the synthesis and remodeling of the matrix. Collagenous proteins compose most of the matrix. H-PRF and CHA contain cytokines, vitamins, or amino acids that are beneficial for collagen formation. TGF-β1 can react with cell membrane receptors and activate the TGF-β signaling pathway, resulting in the transcription of COL1A1. Research has revealed that TGF-β can upregulate the expression of various enzymes in the glycolytic pathway, which is essential for collagen synthesis.^27^ Vitamin C can significantly increase type I collagen fiber formation.^28^ Furthermore, CHA supplies a variety of amino acids that serve as raw materials for collagen formation.

The composition of skin and gingiva is relatively comparable. CHA has recently been widely used in the treatment of aging skin to promote regeneration. As a commercially available injectable gel, CHA can be used clinically without complex clinical trials and drug approvals. PRF is an autogenous biomaterial that provides scaffolds and cytokines for the growth of HGFs. Moreover, oral bacteria initiate the development of gingivitis and periodontitis. In this context, PRF can deliver antimicrobial peptides and obstruct the spread of bacteria, thus protecting gingival health.^29^ CHA-PRF procedure lasts about 40 min, which makes it suitable for clinical periodontal injection treatment. This could possibly avoid surgeries to achieve soft tissue regeneration and alleviate patients’ pain. CHA-PRF exhibited prominent advantages for HGF vitality and function. The combined use of CHA and PRF can provide a convenient and effective method for soft tissue regeneration.

## Conclusions

In this study, the CHA-PRF group exhibited better cell viability, better collagen synthesis ability, and better chemotaxis and migration than the pure CHA or PRF group. The combination of CHA and PRF can provide a superior microenvironment for periodontal or peri-implant soft tissue regeneration.
